# Profile of Bacterial Communities in Copper Mine Tailings Revealed through High-Throughput Sequencing

**DOI:** 10.3390/microorganisms12091820

**Published:** 2024-09-03

**Authors:** Joseline Jiménez-Venegas, Leonardo Zamora-Leiva, Luciano Univaso, Jorge Soto, Yasna Tapia, Manuel Paneque

**Affiliations:** 1 Faculty of Agricultural Sciences, University of Chile, Santa Rosa 11315, La Pintana, Santiago 8820808, Chile; jjimenezve@fen.uchile.cl (J.J.-V.); yasnatapiafernandez@uchile.cl (Y.T.); 2 Master Program in Territorial Management of Natural Resources, University of Chile, Santa Rosa 11315, La Pintana, Santiago 8820808, Chile; 3 Fundación Bionostra Chile Research, Almirante Lynch 1179, San Miguel, Santiago 8920033, Chile; lzamora@bionostra.com (L.Z.-L.); lunivaso@bionostra.com (L.U.); jsoto@bionostra.com (J.S.)

**Keywords:** mine tailing, 16S rRNA, metals, bacterial communities

## Abstract

Mine-tailing dumps are one of the leading sources of environmental degradation, often with public health and ecological consequences. Due to the complex ecosystems generated, they are ideal sites for exploring the bacterial diversity of specially adapted microorganisms. We investigated the concentrations of trace metals in solid copper (Cu) mine tailings from the Ovejería Tailings Dam of the National Copper Corporation of Chile and used high-throughput sequencing techniques to determine the microbial community diversity of the tailings using 16S rRNA gene-based amplicon sequence analysis. The concentrations of the detected metals were highest in the following order: iron (Fe) > Cu > manganese (Mn) > molybdenum (Mo) > lead (Pb) > chromium (Cr) > cadmium (Cd). Furthermore, 16S rRNA gene-based sequence analysis identified 12 phyla, 18 classes, 43 orders, 82 families, and 154 genera at the three sampling points. The phylum Proteobacteria was the most dominant, followed by Chlamydiota, Bacteroidetes, Actinobacteria, and Firmicutes. Genera, such as *Bradyrhizobium*, *Aquabacterium*, *Paracoccus*, *Caulobacter*, *Azospira*, and *Neochlamydia*, showed high relative abundance. These genera are known to possess adaptation mechanisms in high concentrations of metals, such as Cd, Cu, and Pb, along with nitrogen-fixation capacity. In addition to their tolerance to various metals, some of these genera may represent pathogens of amoeba or humans, which contributes to the complexity and resilience of bacterial communities in the studied Cu mining tailings. This study highlights the unique microbial diversity in the Ovejería Tailings Dam, including the discovery of the genus *Neochlamydia*, reported for the first time for heavy metal resistance. This underscores the importance of characterizing mining sites, particularly in Chile, to uncover novel bacterial mechanisms for potential biotechnological applications.

## 1. Introduction

After the extraction of economic metals, the resulting mixtures are deposited as sediments over wide areas, forming distinctive tailing mounds at both active and abandoned mines [1,2]. The composition of mine tailings varies depending on the mineralogy of the ore deposit and the processing methods [3]. Mining tailings from copper (Cu) mining (central zone of Chile), mainly associated with chalcopyrite (CuFeS_2_), can contain a combination of ground rock, sand, clay, chemicals from metallurgical processes, residual metals (mainly copper [Cu], iron [Fe], and molybdenum [Mo]), and minerals (like sulfides, silicates, and oxides) [4,5,6]. Although Cu mining contributes significantly to the global economy [7,8] and is a significant driver of economic growth in many countries [9,10], the high metal content in the residual soil poses significant environmental hazards [11], ranging from soil and water pollution to the disruption of ecosystems and release of potentially harmful substances into the environment [12].

If mining tailings are not properly sealed, they can cause soil and water contamination due to the deposition of metals or other elements [13]. The risks posed by mine tailings are related to environmental factors, such as temperature, wind, and precipitation, which affect the dispersion of metals and other elements from Cu mine tailings [14,15]. Ecologically, metals in mine tailings affect soil microbial diversity and species distribution, promoting the growth of selective dominant species [16], limiting microbial reproduction [17], and diminishing the ecological roles of microbes, such as carbon and nitrogen metabolism [18]. Microbial composition can be affected by various chemical and physical factors in a given habitat, such as pH, metal concentration, temperature, salinity, organic matter, and oxygen content [19,20,21]. Mine tailings, in addition to being a source of metals, contain microorganisms that play key roles in maintaining soil composition and recycling metals; hence, understanding this diversity is crucial for biomining and preventing adverse effects on nearby productive soils [22,23]. Although the vast biodiversity and uncultivable nature of many microorganisms make it challenging to accurately represent the microbial communities in specific ecological niches, metagenomics has revolutionized the study of microbial biodiversity, adaptation to ecological niches, and evolution [24,25,26].

Failures of tailing impoundments can swiftly worsen environmental pollution, as evidenced by incidents in Brazil and Spain where homes were destroyed and water supplies were disrupted [27]. Active tailing sites are particularly prone to failure, posing a greater risk than abandoned or improperly closed ones [1,28]. This highlights the urgent need for strategies to reduce heavy metal concentrations at these locations.

Microbial remediation offers a potential solution by employing urease-producing bacteria, phosphorus-solubilizing bacteria, iron/manganese-oxidizing bacteria, nitrate-reducing bacteria, sulfate-reducing bacteria, and oligotrophic iron-reducing acidophiles [29,30,31]. Microbial-induced precipitation of metal(loid)s as carbonates, phosphates, and sulfides has been documented [32]. For instance, microorganisms that catalyze the reduction of Cr^6+^ to Cr^3+^ and the oxidation of As^3+^ to As^5+^ can reduce the toxicity of chromium and arsenic [33]. Additionally, microbial reduction of As^5+^ and SO_4_^2−^ can facilitate the precipitation of arsenic–sulfide minerals [34].

However, even with state-of-the-art techniques, our understanding of microbial community responses to environmental stresses and contaminants, as well as their role in metal solubilization, remains limited because of the lack of comprehensive geochemical datasets combined with metagenomic sequence data [35]. The high reactivity of sulfides in mine tailings can lead to elevated metal and salinity levels through the coupled weathering of sulfide and carbonate wastes [36]. This creates a hostile environment for microbes, yet a significant number of phyla, such as Proteobacteria, Actinobacteria, Euryarchaeota, Firmicutes, and Nitrospirae, albeit with relatively low diversity compared with that in normal soils, have been detected in mine tailings [28] and have been identified for their potential metal tolerance [37].

Several bacterial genera (*Pseudomonas*, *Bacillus*, *Arthrobacter*, *Cupriavidus*, and *Acinetobacter*) are known for metal resistance mechanisms, such as biosorption, bioaccumulation, enzymatic transformation, and precipitation [38]. Key features of these microorganisms include high tolerance to metals, the ability to form biofilms, the production of extracellular polymeric substances, and the possession of metal-resistant genes [37]. These features enhance their survival in contaminated environments, as well as their efficiency in metal detoxification [39]. Metallotolerant microorganisms can originate from sites with a high metal content, such as mine tailings, industrial waste sites, and other contaminated environments. These environments often harbor indigenous microbial communities that have adapted to high metal concentrations and can be isolated for bioremediation purposes [40,41]. The microbial composition in mine tailings has not been characterized in Chile, a country with great mining activity. In this work, we seek to characterize the bacterial communities present in the Ovejería Tailing Dam, with the aim of identifying bacterial strains with potential biotechnological uses, specifically focused on bioremediation. The adverse conditions (especially heavy metals) present in the tailings probably serve as a selection pressure [42], which makes it possible to find bacteria that not only tolerate but also resist these metals and that can eventually be used as bioremediators.

In this study, we set out to identify the bacterial community present in the Ovejería Tailings Dam to investigate the extreme environment under which these extremophile bacteria survive and to identify which have the ability to withstand high metal contents. To do this, we analyzed samples from three different areas containing copper mining tailings from the Ovejería Dam, belonging to the National Copper Corporation of Chile (CODELCO), located in the central zone of Chile. The metal concentrations of the samples were determined, and high-throughput sequencing techniques were used to examine the microbial communities based on 16S rRNA (gene) amplicon sequencing data, taxonomic identification, and microbial diversity analysis. This is the first time that bacterial communities have been identified in these mining tailings using metagenomic tools. Studies have been carried out in this location seeking to generate alternatives for the remediation of mining tailings once the closure stage has begun. In turn, this is a tailings dam that is located in the capital of Chile and that has nearby towns (Chacabuco, Huechún, Huertos Familiares, Punta Peuco, and Santa Matilde) less than 8 km away, which could be affected in the event of material displacement [43]. Having an adequate closure is essential to prevent possible risks. Elucidating the microbial communities present is a first step in contributing to the circular economy as well as disaster prevention, thus helping to identify future uses of this waste.

Characterizing microbial communities in tailing dams with heavy metal contamination is essential for uncovering novel bioremediation mechanisms. These insights can lead to innovative strategies for mitigating environmental contamination and harnessing microbial potential for biotechnological applications.

## 2. Materials and Methods

### 2.1. Study Area

**Figure 1:**
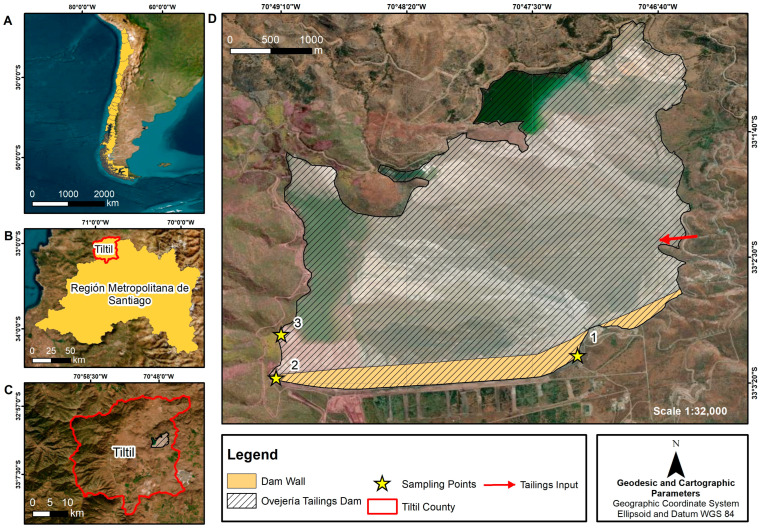
Ovejería Tailings Dam, CODELCO Division. (**A**) Location of Chile in South America. (**B**) Location of Santiago, the capital city of Chile (Metropolitan Region). (**C**) Tiltil County. (**D**) Location of Ovejería Tailings Dam and sampling points (indicated by yellow stars and numbers). In areas where the surface of the tailing dam is hatched, the dam wall is shown in orange. For illustrative purposes, the red arrow indicates one of the operating points where fresh tailings enter into the Ovejería Tailings Dam.

The study area corresponded to the Ovejería Tailings Dam, CODELCO company, Andina Division (UTM coordinates: south: 6342182.19; east: 335223.6; 898.77 m a.s.l.), located in the county of Tiltil, Santiago Metropolitan Region [44] (Figure 1). The mine tailings examined in this study are a solid gray material with a sandy texture; in simple terms, this is a residual ground rock that remains after the Cu extraction process from the mined chalcopyrite mineral (CuFeS_2_) [43]. The NPK (6.55, 0.4, and 9.32 mg kg^−1^, respectively), MO (0.31%), and CEC values reported by Tapia et al. [45] indicate low fertility, which is common in mining tailings. The percentages of sand, silt, and clay are 85%, 13%, and 2%, respectively [45].

### 2.2. Tailing Sampling

Sampling points were related to the accessibility of the tailings. Given the prior authorization of the company in charge of the tailings, samples were taken from the dam wall of the Ovejería Tailings Dam (solid tailing) at a depth of 10 cm. Samples were collected from three points, with three replicates at each point (P1: 33°03′07″ S, 70°47′13″ W; P2: 33°03′18″ S, 70°49′12″ W; P3: 33°03′01″ S, 70°49′10″ W, H19) (Figure 1), in sterile 50 mL tubes. P1 and P2 correspond to points located on the wall of the Tailings Dam, where the thick section of the tailings is deposited. P3 corresponds to a point located in the beach area of the tailings dam. The sector near P1 has previously been physicochemically characterized, where the presence of quartz (50.74%), muscovite (19.04%), albite (16.01%), orthoclase (13.05%), pyrite (0.77%), and clinochlore (0.39%) was reported [39]. Each tube was immediately placed in a sterile, labeled bag. The samples were stored in a container at low temperature (4–8 °C) and transferred to the laboratory, where they were stored at −20 °C until further analysis [46,47].

### 2.3. Physicochemical Parameters of the Mine Tailings

The physicochemical parameters of the samples were determined according to the methods described by Tapia et al. [45].

The pH was determined in an aqueous suspension (1:2.5, *w*/*v*) using Hanna HI 3222 (HANNA Instruments, Woonsocket, RI, USA) equipment, and electrical conductivity was determined in a saturated extract using Hanna HI 4321 equipment (HANNA Instruments, Woonsocket, RI, USA). Organic carbon in solid samples was determined through the oxidation method using H_2_SO_4_ and Na_2_Cr_2_O_7_, and carbon was determined using a colorimetric method, with measurements compared against a sucrose standard curve at 600 nm [48] using a Hach DR5000 spectrophotometer (Hach, Loveland, CO, USA). Total organic matter (OM) was calculated as OM (%) = 1.72 × total organic carbon (%).

The cation exchange capacity (CEC) of the material was determined using Na^+^ acetate and NH_4_^+^ acetate at pH 7.0 [49], and the concentration of Na was measured through atomic absorption spectrophotometry (AAS) using a PerkinElmer PinAcle 500 instrument (PerkinElmer, Boston, MA, USA).

Available nitrogen (N) was determined through extraction with KCl, followed by distillation and NH_4_ titration [50]. Available phosphorus was extracted with sodium hydrogen carbonate (0.5 M, pH 8.5) using Olsen’s method [51]. SO_4_^−^ was determined in the saturated extract using a colorimetric method with BaCrO_4_ [52]. Available sulfur (S) was determined through extraction with 0.01 M Ca(H_2_PO_4_)_2_ and turbidimetry [53].

For the determination of the total concentration of the chemical elements cadmium (Cd), chromium (Cr), Cu, Fe, manganese (Mn), Mo, and lead (Pb), the samples were digested with a strong acid (HCl [4 mL]–HNO_3_ [6 mL]) [54], and the concentration of metals was determined through AAS using Perkin Elmer FIAS 100 equipment (detection limit: 0.01 μg L^−1^). For the determination of micronutrients (available metals: Cd, Cu, Fe, Mn, Ni, and Zn), an extraction was performed with DTPA-CaCl_2_-TEA (DTPA solution) at pH 7.3. Then, 10 g of tailings was weighed, and 20 mL of DTPA solution was added, stirred for exactly 2 h, and then filtered through a Whatman N°42 filter. The concentrations of Cd, Cu, Fe, and Mn were determined through AAS [55]. For the determination of the micronutrients As, Mo, and Cr, a different methodology was used based on the sequential extraction of arsenic by Ascar et al. [56] and Moreno-Jiménez et al. [57]. First, 1.00 g of dry, finely crushed, sieved tailings (0.200 nm) was weighed, and then 25 mL of NH_42_SO_4_ (0.05 M) was added, and the mixture was shaken for 4 h and finally centrifuged at 3000 rpm (2600× *g*) for 10 min. The concentrations of Cr and Mo were read through ASS.

All analyses were conducted in triplicate. Element concentrations are expressed in mg kg^−1^ dry matter.

### 2.4. DNA Extraction and Sequencing

DNA was extracted as described through phosphate buffer extraction according to Guerra et al. [58]. In sterile 2 mL tubes, 0.5 g of tailings (*n* = 3) was weighed, and 500 μL of phosphate buffer (Na_2_HPO_4_ 1 M, KH_2_PO_4_ 1 M, pH 7.2, with 0.5% (*w*/*v*) sodium dodecyl sulfate) was added. The samples were shaken at 1500 rpm for 10 min using a Vortex Genie 2 apparatus (Scientific Industries, Bohemia, NY, USA), followed by centrifugation at 8000 rpm (6800× *g*) for 1 min in a 5810 R centrifuge (Eppendorf, Hamburg, Germany). Subsequently, 90 μL of the supernatant was transferred to a fresh 2 mL tube and diluted 1:10 by adding 810 µL of ddH_2_O, followed by the addition of 900 μL of phenol [59]. The samples were shaken through inversion and centrifuged at 8000 rpm (6800× *g*) for 10 min, and an 800 μL aliquot of the supernatant was transferred to a new 2 mL tube. Then, 800 μL of chloroform–isoamyl alcohol (24:1 [*v*/*v*]) was added to each tube and shaken through inversion, after which the samples were incubated at −20 °C for 10 min. After incubation, the samples were centrifuged at 8000 rpm (6800× *g*) for 10 min. An 800 μL aliquot of the aqueous phase was transferred, and an equal volume of chloroform–isoamyl alcohol was added. After centrifugation, a 600 μL aliquot of the aqueous phase was transferred to a 1.5 mL tube, to which 200 μL of 30% (*w*/*v*) polyethylene glycol (PEG 8000) and 100 μL of 5 M NaCl were added. The samples were vortexed for 10 s and then incubated at room temperature for 20 min. Subsequently, the tubes were centrifuged at 14,000 rpm (20,800× *g*) for 20 min to precipitate the DNA. The supernatant was discarded, and the precipitated DNA was washed with 500 μL of 80% (*v*/*v*) EtOH and centrifuged at 14,000 rpm (20,800× *g*) for 5 min. This washing step was performed in duplicate. To remove the EtOH from the precipitated DNA, the DNA was dried at room temperature for 30 min and resuspended in 31.5 μL of TE buffer (10 mM Tris, 1 mM EDTA, pH 8.0) and 3.5 μL of MgCl_2_, resulting in a final concentration of 3.5 mM Mg^2+^. The DNA was stored at −20 °C until further use.

The DNA was quantified using a QUBIT™ 4 fluorometer (Thermo Fisher Scientific, Waltham, MA, USA). The integrity of the 16S rRNA region was confirmed through amplification (~1500 bp) via conventional polymerase chain reaction (PCR) with the primers 27F (5′-AGAGAGTTTGATCCTGGCTCAG-3′) and 1492R (5′-GGTTACCTTGTTACGACTT-3′), conducted using a ESCO Swift MaxPro thermal cycler (Thermo Fisher Scientific). The DNA quality of the samples was verified using 1% agarose gel electrophoresis with the SYBR™ Safe DNA gel stain (Thermo Fisher Scientific) coupled to a 1500 bp molecular weight marker. Electrophoresis was performed at 100 V for 30 min and visualized in a transilluminator chamber.

The preparation of genomic libraries and subsequent sequencing of the V3–V4 variable regions of the 16S rRNA gene was performed by Macrogen Inc. (Seoul, South Korea) using a MiSeq^®^ sequencer (Illumina, San Diego, CA, USA). To construct the libraries, adapters were added, and bacteria-specific primers were used to amplify the V3–V4 hypervariable regions (341F: CCTACGGGGGNGGCWGCAG and 805R: GACTACHACHVGGGGGTATCTAATCC). Short PCR cycles were used to generate 16S rRNA amplicons, which were subsequently validated using the Bioanalyzer 2100 system (Agilent Technologies, Santa Clara, CA, USA). Sequencing was performed in a sense and an antisense manner with a 2 × 300 bp configuration.

### 2.5. Bioinformatic Analysis

FASTQ files generated by the sequencing companies were processed using Qiime v2023.9.1 [60]. The quality of the reads was evaluated using FastQC v0.11.9. Based on this evaluation, adapters were removed, and the lengths of the forward and reverse sequences were trimmed to 280 and 243 bases, respectively, using the Dada2 denoise-paired plugin. Quality was maintained using PHRED 20 [61]. Taxonomic assignment was performed with the classify-sklearn plugin using the SILVA database version 132 (https://www.arb-silva.de; accessed on 3 June 2024). New unified sequences (operational taxonomic units [OTUs] with 99% identity) were aligned using MAFFT [62], and maximum likelihood was inferred using FastTree [63].

The abundance, taxonomy, phylogeny, and metadata of the OTUs were integrated into a phyloseq object for subsequent analyses using the Phyloseq v1.46.0 package (PhyloSeq [64]) in R v4.3.2 (https://www.r-project.org; accessed on 3 June 2024). Quality-control filters described by Callahan et al. [65] were used; samples with <1000 reads were excluded; unassigned OTUs were removed; the mean number of reads per taxon was >1 × 10^−5^; and OTUs that were not observed more than twice in at least 10% of the samples were excluded. Then, the phyloseq object was used for the calculation of α- and β-diversity and the construction of relative abundance plots with the Microeco v1.4.0 package. OTUs were transformed into proportions, and relative abundance was used to measure the composition of prokaryotic communities at the phylum, family, and genus taxonomic levels. α-diversity was calculated using different indices, including the Chao1 (estimate of richness by species) [66], Pielou (estimate of abundance and uniformity of OTUs in the samples) [67], Shannon (entropic information on the abundance of the OTUs observed) [68], and Simpson (1-dominance) indices [69].

For determining β-diversity, all samples were compared according to the sampling points using six matrices (Bray–Curtis, Canberra, Jaccard, JSD, Unifrac, and wUnifrac). The presence of each genus at every point was correlated with the metal concentration using a Spearman correlation. A Spearman correlation analysis was performed and visualized using the Microeco package v1.4.0 with the cal_cor function. The correlations between the relative abundances of the 27 common genera and the concentrations of seven metals (total and available) were calculated. The resulting correlation matrix was visualized using the plot_cor function.

A Venn diagram was made by reviewing those genera that were uniquely found at each sampled point, those that were shared between two points, and the common ones that were found at all three points.

## 3. Results

### 3.1. Chemical Characterization

The Ovejería Tailings Dam was neutral to slightly acidic and slightly saline, with very low organic matter content (<0.17% at three points) (Table 1).

Both P1 and P2 showed no vegetation or any ground cover. However, P3, which was closer to the tailing water beach, showed certain tree species, as well birds along the beach shore, potentially contributing to organic matter in this area. A sandy texture was observed at all three points, with P1 having the highest humidity, which can be attributed to regular water application in various sectors of the wall to prevent the spread of tailing dust.

Because the fresh tailings enter through a sector far from the wall and the sampled points (red arrow, Figure 1), and given the way the Ovejería Tailings Dam is built (“downstream”), it can be understood that although we were not working with fresh tailings, we were not analyzing the oldest tailings, either [70].

Table 1 shows the concentration of total metals at P1, P2, and P3, highlighting Cu (2488 ± 258, 2986 ± 102, and 2911 ± 806 mg kg^−1^, respectively) and Mo (145.6 ± 8.90, 268.8 ± 84.1, and 169.03 ± 21.8 mg kg^−1^, respectively) and the concentrations of the available metals (accessible to be absorbed by living organisms, either through absorption by plants, microorganisms, or animals), highlighting Cu (67.1 ± 2.9, 69.8 ± 4.1, and 80.4 ± 4.3 mg kg^−1^, respectively) and Mn (6.69 ± 0.2, 1.25 ± 0.08, and 10.5 ± 1.14 mg kg^−1^, respectively) as the metals with the highest concentrations. Available Mo was not detected at P1 or P3, but it was detected at P2 at a low concentration (0.55 mg kg^−1^).

### 3.2. Bacterial Community Diversity and Distribution

To determine the diversity and distribution of the bacterial communities, 522 bacterial OTUs corresponding to 16S rRNA gene sequences (P1: 163; P2: 231; P3: 253) were analyzed after applying quality filters (239,138 total reads; P1: 79,254; P2: 70,166; P3: 89,718). These OTUs reflect the microbial diversity in the tailings and represent 12 phyla, 18 classes, 43 orders, 82 families, and 154 genera.

**Figure 2:**
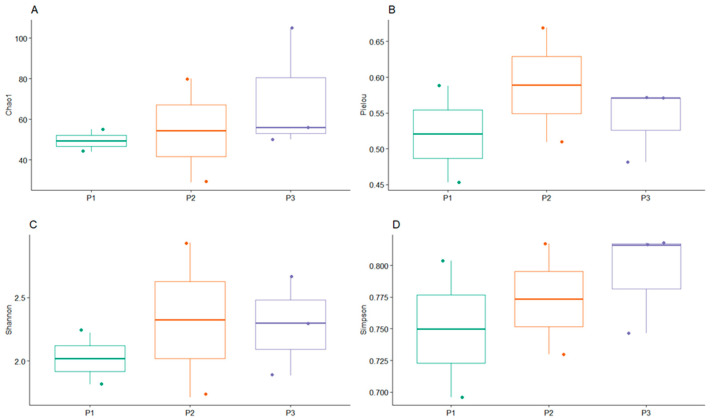
Alpha-diversity plots among the sample points in the Ovejería Tailings Dam. (**A**) Chao1, (**B**) Pielou, (**C**) Shannon, and (**D**) Simpson indices (*p* < 0.05).

Of the four diversity indices applied, no significant differences were observed between the sampled sites (Figure 2).

**Figure 3:**
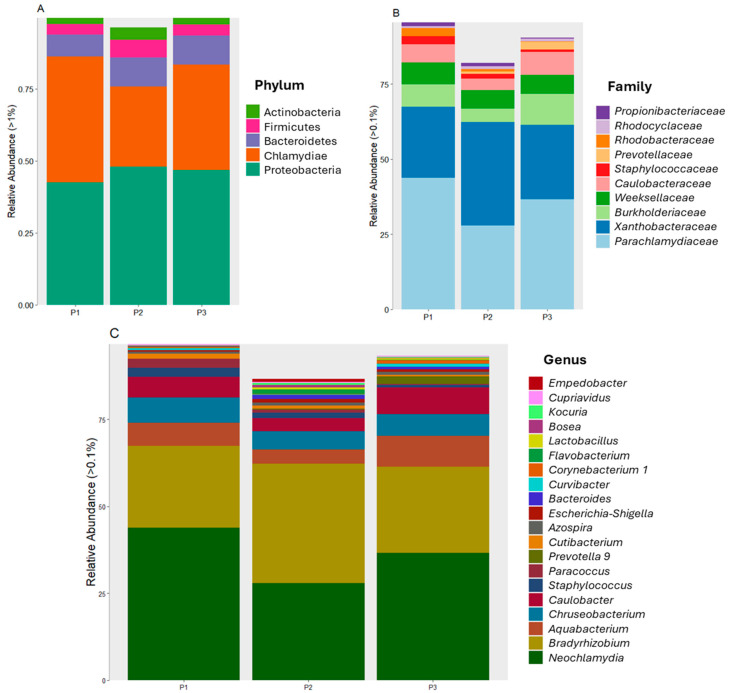
Relative abundances of bacteria per sampled point (**A**) at the phylum level, (**B**) at the family level, and (**C**) at the genus level.

Proteobacteria was the most dominant phylum, with relative abundances of 42.4%, 48%, and 46.7% at P1, P2, and P3, respectively, showing a similar abundance between P2 and P3. The second most dominant phylum was Chlamydiota, with relative abundances of 43.8%, 27.9%, and 36.7% at P1, P2, and P3, respectively, with a slight decrease at P2. Bacteroidetes, as well as Proteobacteria, showed a similar abundance at P2 and P3 (10.0% and 10.1%, respectively), which was lower at P1 (7.6%). Firmicutes, together with the phylum Actinobacteria, presented a similar abundance between P1 and P2 (Firmicutes: P1: 3.5%; P2: 3.8%; Actinobacteria: P1: 2.3%; P2: 2.4%) but a higher abundance at P2 (6.1% and 4.4%, respectively, for both phyla). Cyanobacteria had the lowest abundances (1.8% at P2 and only 0.01% at P3, with no presence at P1). The other phyla accounted for 0.29%, 1.84%, and 0.28% of the bacteria at P1, P2, and P3, respectively (Figure 3A).

At the family level, it was observed that the families *Parachlamydiaceae* (P1: 43.8%; P2: 27.9%; P3: 36.7%, the most abundant) and Caulobacteraceae (P1: 6.1%; P2: 4.0%; P3: 7.7%) had a greater abundance at P1 and P3 than at P2. The second most abundant family was *Xanthobacteraceae* (P1: 23.5%; P2: 34.4%; P3: 24.7%), which, unlike the previous two, had a greater abundance at P2. With regard to the *Burkholderiaceae* family (P1: 7.5%; P2: 4.5%; P3: 10.3%), its presence at P3 was over twice that at P2. The fourth most abundant family was *Weeksellaceae* (P1: 7.3%; P2: 6.1%; P3: 6.3%), which showed similar relative abundances at the three sampled points, unlike the other most abundant families, which showed non-uniform abundance at the three points. The remaining predominant families had an average relative abundance of 4% among the sampled points, and the remaining families present in the tailings had relative abundances of 6.4%, 20%, and 10.82% at P1, P2, and P3, respectively. P2 exhibited a relative abundance of the following bacteria: *Enterobacteriaceae* (1.2%), *Beijerinckiaceae* (1.1%), *Lachnospiraceae* (1.2%), *Bacteroidaceae* (1.2%), *Flavobacteriaceae* (1.3%), and *Nostocaceae* (1.1%) (Figure 3B).

At the genus level (Figure 3C), eight genera were the most abundant: *Neochlamydia*, *Bradyrhizobium*, *Aquabacterium*, *Chryseobacterium*, *Caulobacter*, *Staphylococcus*, *Paracoccus*, and *Cutibacterium* (>86.0% relative abundance). *Neochlamydia* (P1: 43.8%; P2: 27.9%; P3: 36.7%) had a higher relative abundance at P1 and P3, unlike *Bradyrhizodium* (P1: 23.5%; P2: 34.4%; P3: 24.7%), which had a higher abundance at P2. *Aquabacterium* (P1: 6.6%; P2: 4.0%; P3: 8.8%) and *Caulobacter* (P1: 5.9%; P2: 3.8%; P3: 7.6%) had the highest relative abundances at P3, whereas *Chryseobacterium*, *Staphylococcus*, *Paracoccus*, and *Cutibacterium* had the highest relative abundances at P1 (7.3%, 2.7%, 2.6%, and 1.4%, respectively).

### 3.3. Biodiversity Analysis of Bacteria Associated with Mining Tailings across Three Zones

**Figure 4:**
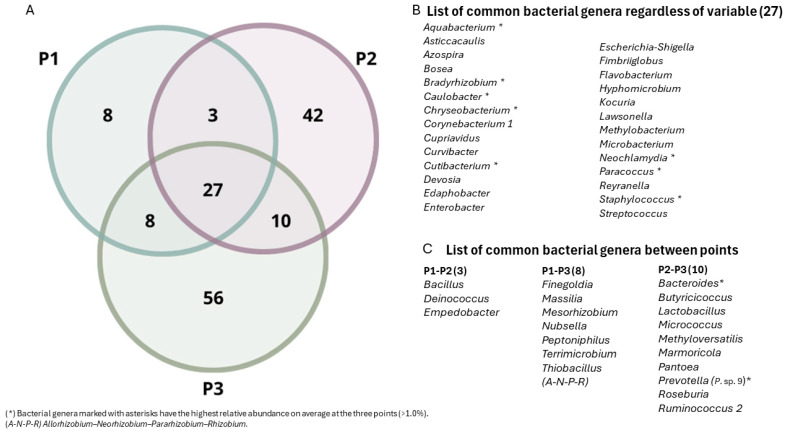
(**A**) Venn diagram showing the number of common bacterial genera between three different zones belonging to the Ovejería Tailings Dam. (**B**) List of common bacterial genera regardless of the variable. (**C**) List of common bacterial genera between points. (*) Bacterial genera marked with asterisks have the highest relative abundance on average at the three points (>1.0%). (A-N-P-R) *Allorhizobium–Neorhizobium–Pararhizobium–Rhizobium*.

Twenty-seven bacterial genera were the most commonly observed in the samples obtained from three different tailing zones (P1, P2, and P3) (Figure 4B; Data S1). Importantly, the shared bacterial genera had the highest relative abundance at each location (>75%).

The Venn diagram (Figure 4A) at the genus level showed that eight genera were found only in the P1 sample, accounting for 0.47% of the relative abundance at this sampling point. The P2 sample contained 42 unique genera with a relative abundance of 10.54%, and the P3 sample contained 56 unique genera with a relative abundance of 4.25%. Moreover, the P1 sample shared three genera with the P2 sample and eight with the P3 sample, whereas the P2 and P3 samples shared ten genera.

**Figure 5:**
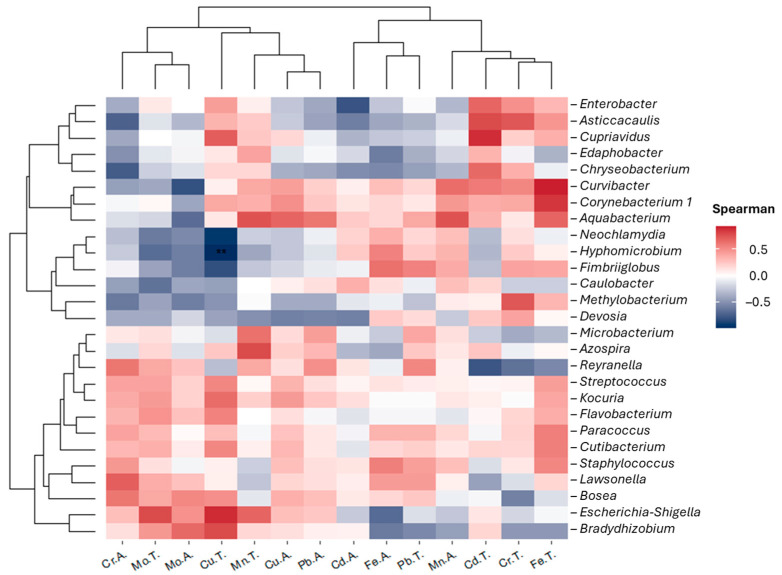
Spearman correlation between the relative abundances of 27 common genera and the concentrations of seven metals (total and available concentrations) across the three sampling points (P1, P2, and P3). The X-axis represents metals, while the Y-axis represents genera. Color intensity indicates the strength of the correlation, with positive correlations in red and negative correlations in blue. Significant correlations are highlighted with (*).

Figure 5 presents a heatmap of Spearman correlations between the relative abundances of 27 common bacterial genera and the concentrations of metal calculated in this study (total and available) at the three sampling points (P1, P2, P3).

*Hyphomicrobium* presented a significant negative correlation with the presence of total Cu; as the presence of total Cu increased, that of *Hyphomicrobium* decreased. Furthermore, *Neochlamydia* presented a negative correlation with the concentration of total Cu; however, the correlation was not significant.

*Curvibacter*, *Corynebacterium 1*, and *Aquabacterium* presented a positive correlation with the concentration of total Fe; an increased relative abundance of these genera was observed with the increasing presence of total Fe.

In the case of the second most abundant genus, *Bradyrhizobium*, a slight positive correlation was observed with the concentrations of total Cu, available Mo, and total Mo, and a negative correlation was observed with available Fe and total Pb.

## 4. Discussion

### 4.1. Chemical Parameters of the Tailing Samples

Our results showed significant variations in metal concentrations at the three sampling points, with the following order of magnitude: Fe > Cu > Mn > Mo > Pb > Cr > Cd. Fe is a commonly found metal in mine tailings; hence, high concentrations of total Fe are expected and associated with reactions that generate acidity [71]. The total concentration of Cu found in other mine tailings ranges from 1.0 to 750 mg kg^−1^ [72], which was exceeded at all three sampled points, with P2 revealing the highest concentrations, averaging 2986 ± 102 mg kg^−1^. Mo concentrations also varied between the points, being highest at P2 (268.8 ± 84.1 mg kg^−1^) and lower at P1 (145.6 ± 8.90 mg kg^−1^) and P3 (169.03 ± 21.8 mg kg^−1^), with all three points falling within the range of Mo concentration found in other mine tailings (100–800 mg kg^−1^) [45]. These elevated concentrations of Cu and Mo may be associated with the mineralogy of the mining site that generates the tailings and specific geological conditions of the sampling area. Tapia et al. [43] investigated the same tailings and noted that as a residual ground rock obtained after the extraction of chalcopyrite (CuFeS_2_), the high concentration of Cu can be attributed to this mineral. The formation of Acid Mine Drainage (AMD) was not observed at the sampled points, which is associated with the presence of chalcopyrite in the tailings studied, where values of 1.0–1.7% are considered low [54], which would help with the prevention of AMD generation [45]. The pH value of the tailing samples indicates that there is no oxidative process that generates acidity, as is common in mining tailings [71]. Keeping track of AMD generation is important because its acidic pH can cause the leaching of metals, increasing the risk of contaminating groundwater [43].

A higher concentration of available Cu than that previously reported by Tapia et al. [45] was observed (67.1 ± 2.9 to 80.4 ± 4.3), but considering the Kelly index for contaminated soils, it was still below the range of “uncontaminated soil” [73].

### 4.2. Insights into the Variability and Spatial Distribution of Bacterial Communities

At the phylum level, a high abundance of Proteobacteria (Pseudomonadota) was observed in the P1, P2, and P3 samples (42.4%, 48%, and 51%, respectively). These results were consistent with those of studies conducted by Li et al. [74] and Gupta et al. [75] in other Cu tailings. Li et al. [74] reported that the relative abundance of Proteobacteria was 25%, with high concentrations of total Cu (1280 mg kg^−1^, close to the average found in the Ovejería tailing, ~2000 mg kg^−1^). However, the availability of this metal (available Cu: 0.12 mg kg^−1^) was much lower than that reported in the present study (~72.4 mg kg^−1^). Gupta et al. [75] found that in an environment with lower total Cu concentrations between 274 and 509 mg kg^−1^, Proteobacteria was the most abundant phylum, with 55% and 45% relative abundance, respectively. Proteobacteria are known for their metal resistance and detoxification mechanisms, such as biosorption and bioaccumulation, which allow them to thrive in soils contaminated with Cu, Zn, and Pb [76,77,78]. The role played by these microorganisms in the Ovejería Tailings Dam has not been studied in depth, but given that they at least survive in an environment with high metal content, they may hold potential as a biotechnological tool that would allow for a possible remediation of environments with high metal content.

Within the phylum Chlamydiota (the second most abundant phylum), the genus *Neochlamydia* had the highest relative abundance (P1: 43.8%; P2: 27.9%; P3. 36.7%). It is important to note that although this genus exhibits one of the highest relative abundances, the literature on its tolerance to metals is limited. Therefore, this study may be among the first to report such a high abundance of this genus in environments with elevated Cu concentrations. Environments in which this genus has been reported in the literature are primarily associated with water. This genus has been reported in a study of activated sludge from wastewater, suggesting that this environment is a reservoir for environmental Chlamydiae [79].

Within the Proteobacteria phylum, the second most abundant genus was *Bradyrhizobium* (P1: 23.5%; P2: 34.4%; P3: 24.7%). This genus is typically associated with nitrogen fixation in the presence of legumes [80,81]. No vegetation was observed near P2. However, P3 showed vegetation, with a slight decrease in the abundance of *Bradyrhizobium* (P3: 24.7%). *Acacia caven* could be observed with the naked eye a couple of meters from the sampling point, a distance that may not necessarily influence the microbial composition of the sampling point. P1 (23.5%) showed similar relative *Bradyrhizobium* abundance to that of P3, with no vegetation present, as at P2. This supports findings that the *Bradyrhizobium* genus has been widely associated with the rhizosphere of legumes, but free-living populations of this genus have also been observed without the possibility of forming nodules with plants [82,83]. The sampled points do not have vegetation covering the soil, so this would reinforce the idea that these bacteria are capable of surviving freely [84,85]. Although this genus has been associated with nitrogen fixation, an in situ study conducted by Dary et al. [80], wherein *Lupinus luteus* inoculated with *Bradyrhizobium* sp. 750 was used to phytostabilize soils contaminated with mining spills containing high concentrations of Cu, Cd, and Pb, resulted in an increase in plant biomass. This is consistent with the findings of Seneviratne et al. [86], who demonstrated the ability of this genus to promote the growth of plants found in environments with high levels of available metals, such as Cu, Ni, and Pb. Having bacteria with the ability to promote plant growth, fix nitrogen, and tolerate high concentrations of metals represents an opportunity to bioremediate the high concentrations of metals present, with a joint work between accumulating plants and bacteria that allows their better development in these environments. This genus has also been previously reported by Gerrity et al. [87], who studied ozone-biofiltration systems in which the presence of the genera *Neochlamydia* and *Firmicutes* was also obtained. The joint work of more than one bacterial genus allows us to understand the complexity in which these environments develop, with each genus having a role that allows for the development of other genera.

The genus *Aquabacterium* was the third most abundant genus (P1: 6.6%; P2: 4.0%; P3: 8.8%), with the highest abundance at P3. This genus has been reported with certain pathogenic species in wastewater that had high contents of Cu, Zn, and certain antibiotics [88]. It has been reported to tolerate high concentrations of metals, as do *Bradyrhizobium* species. While it is expected to be associated with aquatic environments, it has been reported in agricultural soils in Thailand with the following metal concentration gradient: Fe > Mn > Zn > Pb > Ni > Cu > Cd. Total Fe concentrations reached maximums of 410 mg kg^−1^ and total Mn reached a concentration of 59.9 mg kg^−1^ [89]. The results of the present study showed Fe and Mn concentrations more than five times higher than those reported by Kroeksakul et al. [89] (total Fe ~30,000 mg kg^−1^; total Mn 268.6 mg kg^−1^), but it is still possible to observe a high relative abundance of the genus *Aquabacterium* in P3 (8.8%).

Within the phylum Proteobacteria, the genus *Paracoccus* was detected, with greater abundance in P1 (2.6%) than in P2 and P3 (0.9% and 0.2%, respectively). This genus has been reported to exhibit Cu bioleaching through S oxidation, thereby promoting Cu bioremediation [79]. Our results indicate a greater presence of sulfates in the P1 sample, which could support the presence of this genus at this point, corroborating what was reported by Andreazza et al. [90]. *Paracoccus* is also recognized for its ability to perform aerobic denitrification [91,92], which has been associated with a reduction in Cu^2+^ [93].

The genera *Aquabacterium*, *Bradyrhizobium*, and *Paracoccus* at each point were correlated with processes associated with the nitrogen cycle. However, *Aquabacterium* and *Paracoccus* stood out, as they have previously been linked to denitrification and shown to influence Fe and Cu oxidation, respectively [93,94]. These genera revealed a positive correlation, without significant differences, with the presence of total Fe (Figure 5). This implies that as the concentration of total Fe increases, so does the relative abundance of the genera *Aquabacterium* and *Paracoccus*.

The genus *Azospira* has been studied mainly in aquatic environments. Su et al. [95] reported the presence of Cu (II) in wastewater contaminated with aniline at concentrations of 3–10 mg/L. Its presence is an important factor for aniline degradation and nitrogen removal [95]. Shi et al. [96] reported the use of several genera of the phylum Proteobacteria, such as *Sphingomonas*, *Azospira*, and *Cupriavidus*, in the construction of biocathode microbial electrolytic cells for the removal of Cu, Pb, Zn, and Cd at initial concentrations of 20 mg/L, achieving favorable results with Cu and Pb removal of approximately 98%.

The genus *Chryseobacterium* (phylum Bacteroidota), the average relative abundance of which at the three sampling points was 6.3%, can be found in various environments, including soil, freshwater, and marine environments [97]. Although most environmental species have not been studied in depth, Majewska et al. [98] studied a strain of this genus and assessed its ability to tolerate higher Cd concentrations (100 mg kg^−1^), low temperatures (8 °C), and 2% salinity. Additionally, this genus has been observed to be tolerant to Cu and antibiotics, being able to withstand concentrations of up to ~1000 mg kg^−1^ [99]. Compared to the results of the present study, it is evident that this genus persists despite high metal concentrations, particularly at elevated Cu, Mn, and Mo concentrations.

The genus *Chryseobacterium* shares a previously described characteristic with the genera *Bradyrhizobium* and *Caulobacter* [83,98,100]. These genera have been reported to produce indole acetic acid (IAA), one of the most important auxin-type plant hormones [101]. IAA is secreted by both soil microbes and plant roots. The combination of bacterial and endogenous IAA from plants stimulates plant cell proliferation [102,103]. Identifying these genera in these environments (mine tailings) can offer insights into the conditions that allow them to thrive and reveal their use in biotechnological applications.

The genus *Caulobacter* (Proteobacteria) had relative abundances ranging from 3.8% in P2 to 5.9% and 7.6% in P1 and P3, respectively. Its abundance in an environment like the one we investigated is not surprising; this genus has been isolated from diverse environments, including deep underground sediments and gold mines [104,105,106]. Additionally, species of this genus have been studied under Cu stress at concentrations between 0 and 200 µM [107].

In the present study, limited information was found on the genus *Prevotella 9* (Bacteroidota), which was identified only in P2 and P3, with relative abundances of 0.18% and 2.3%, respectively. This genus has been reported to exist in soils with high metal contents [108], where it has shown the ability to reduce concentrations of metals, such as Cd, Pb, Cr, Zn, Fe, Cu, and Se.

Within the phylum Actinobacteria, the genus *Cutibacterium* was found to have a relatively low abundance, being more dominant in P1 (1.4%) than in P2 (0.99%) and P3 (0.34%). This genus has been associated with pathologies, such as acne, but it has also been shown to have important functions in skin health and cutaneous microbiota [109,110,111]. Interestingly, this genus is found in environments like that studied here. However, a literature review revealed that it has also been found in oil fields with high Cu and Pb contents (~3812 mg kg^−1^ and ~12,432 mg kg^−1^, respectively), basic pH (pH 10.44), and high NaCl content (50 g/L) [112].

The genera *Cutibacterium*, *Bradyrhizobium*, and *Chryseobacterium* have been investigated in studies examining the effects of NaCl content on these organisms [74,89,101]. In addition to metal tolerance (Cd, Cu, and Pb), *Cutibacterium* and *Prevotella 9* have been associated with oil tolerance [82,98,113].

Within the phylum Firmicutes, relative abundances of the genus *Staphylococcus* in P1, P2, and P3 were 2.7%, 1.6%, and 0.8%, respectively. *Staphylococcus* species have been described as pathogenic and metallotolerant in the human urinary tract, particularly towards Cu and Zn. Studies have reported its resistance to As and Cd, although the resistance was strain-dependent [114]. *Staphylococcus* has been reported to be metallotolerant, being able to withstand total Cu concentrations higher than 1000 mg kg^−1^ [115], so the values obtained in the present study demonstrate the ability of this genus to withstand concentrations close to 2000 mg kg^−1^. Its relationship with Cu tolerance was also studied by Zapotoczna et al. [116], who detailed the mechanisms used by species of this genus to resist death due to high Cu concentrations. They suggested that the gain of mobile genetic elements that carry genes for Cu hypertolerance contributes to the evolution of strains of this genus, which are better-equipped to resist death caused by immune cells of the host in which they live.

### 4.3. Possible Physical Locations Where Bacterial Communities Can Be Found

The bacterial communities found in mining tailings can be distributed based on the physical and chemical characteristics of the environment and the abilities of the bacteria to develop in these environments [117,118]. The predominant minerals in the tailings, such as quartz, muscovite, albite, orthoclase, pyrite, and clonochlore, as well as their sandy texture, offer diverse surfaces and microenvironments that bacteria may be able to colonize [1,119]. The environments that bacteria can colonize include (i) the surfaces of minerals, with the generation of biofilms [120,121]; (ii) pores and fissures [122]; (iii) areas with the presence of pyrite (FeS_2_) [123]; (iv) contact zones with water [124]; and (v) water–mineral interfaces [125].

There are certain bacteria with the ability to generate biofilms, which implies that the bacteria adhere to a particular surface and surround themselves with a matrix of extracellular polymeric substances (EPSs). This matrix provides protection and stability to the bacteria, allowing them to survive in adverse conditions [126]. Genera, such as *Neochlamydia*, *Staphylococcus*, and *Caulobacter*, can form biofilms, which generate a protected environment for other, less-resistant bacteria [127,128,129,130]. Species of the genera *Staphylococcus* and *Microbacterium* have been described with the ability to generate biofilms in the pores found in mining tailings and that have high Cu and Fe contents [127]. In particular, the *Caulobacter* genus, given its curvature, can better colonize surfaces and, at the same time, withstand flows that can displace other bacteria [131]. Like *Bradyrhizobium*, the *Azospira* genus also has the ability to fix nitrogen [132]. In the presence of clay minerals and iron oxides, the generation of biofilms by the *Bacillus* genus has been reported by Ma et al. [133]. In tailings with the presence of copper sulfides, such as chalcocite, digenite, and covellite with bornite and chalcopyrite, the generation of siderophores in the absence of iron by the genera *Bacillus* and *Microbacterium* has been studied [133]. The presence of siderophores is significant because they facilitate the mobilization of ferric ions from insoluble compounds [134]. Organic ligands, such as siderophores, can also strongly influence the dissolution of minerals. Siderophores are capable of binding metals, such as magnesium, Mn, Cr (III), and gallium (III), and radionuclides, such as plutonium (IV) [135].

The *Aquabacterium* genus has the ability to generate biofilms together with certain genera, such as *Dechloromonas*, in the Deilmann Tailings Management Facility (DTMF) water columns that cover the tailings from uranium mines (pH = 7.0), where its greatest abundance was found at approximately 27 and 41 m [136]. *Aquabacterium* has a good, efficient denitrification performance, and it can use ferrous ions (Fe^2+^) as an additional electron donor for complete denitrification in an aqueous environment [137]. This genus has generally been reported in aquatic environments, and although the mining tailings we worked with here have a high percentage of sand and may not retain water for a long time [138], the presence of this genus in the three sampled points is not surprising, as P1 and P2 receive frequent wetting to avoid the dispersion of particles produced by wind erosion. Moreover, because of the proximity of P3 to the tailings dam beach, it is understandable that this point frequently receives contributions from water coming from the same tailings dam, explaining why we could find this genus in greater abundance at P3. In turn, the relationship of this genus with the presence of Cu and Fe was studied, and its greatest abundance was found in P3, where we found the highest concentration of Cu and Fe [94]. *Chryseobacterium* is a genus that can be found in drinking water, generating biofilms [139], and, as with *Aquabacterium*, although it is mostly associated with aquatic environments, given the treatments that are frequently performed on the tailings, it is possible to have traces of these bacteria in the samples analyzed. *Paracoccus* is a non-motile, Gram-negative, bacterial genus that forms a very thin and unique biofilm, unlike other bacterial genera that are characterized by generating biofilms of considerable thickness that can be observed with the naked eye [140]. This genus, in addition to the possibility of generating biofilms, has been studied for its ability to generate siderophores and possess antifungal properties [141].

The dynamics of bacterial communities present in mine tailings from copper extraction through flotation have not yet been studied in depth. Hu et al. [142] studied the ability of bacteria to grow freely or attached to particles in an aquatic environment, revealing that suspended particles and carbon resources promote the similarity between these fractions. In addition, Proteobacteria was found to be one of the most abundant phyla related to the ability to adhere to the surface of suspended particles [142], which added to the evidence found regarding the bacterial genera identified in this study that have the ability to generate biofilms. Due to the characteristics of the minerals present in these tailings, it is possible that these bacteria not only attach to particles but also generate biofilms to improve their chances of survival. The results of the present study allow, as a first step, for the identification of which bacteria have the capacity to develop in these environments. Given the characteristics previously reported, this provide an idea of the mechanisms they use to operate, which could be used as a potential tool when wanting to bioremediate environments with high metal content.

### 4.4. Venn Diagram and Associated Genera

The above-described genera were mostly found at all three sampling points, with a relative abundance exceeding 75%. However, as observed in the Venn diagram (Figure 4), the observations at P2 were particularly notable. Although P2 had a lower number of unique genera (42) than P3 (56), it stood out because these genera had the highest relative abundance (10.54%). The genera with the highest relative abundance that were found exclusively in P2 included genera like *Lachnospiraceae UCG-004*, *Alistipes*, *Tetrasphaera*, *Lysinibacillus*, *Cylindrospermopsis CRJ1*, *Prosthecobacter*, *Pirellula*, *Planomicrobium*, *Pullulanibacillus*, and *Prevotella 7.*

These genera have been previously reported with certain similarities. *Lachnospiraceae*, *Alistipes*, and *Provetella* sp. 7 are mainly associated with the microbiota that can be found in humans [143,144,145]. They may be generators of diseases or, in the case of *Alistipes*, a generator of colorectal cancer or even a protector against cardiovascular diseases or liver fibrosis [144]. On the other hand, it is observed that *Tetrasphaera* has been reported as a phosphorus-remover, which has been studied with a focus on wastewater treatment plants [146]. The presence of *Prosthecobacter* and *Planomicrobium* in low-nutrient and low-temperature environments has been previously reported, so it is not surprising that they can develop in these mine tailings [147,148]. In turn, *Lysinibacillus*, *Cylindrospermopsis*, *Pirellula*, and *Pullulanibacillus* have been related to mining environments, which have been reported as remediators, generators of organic exudates to transport Cu, acidophiles, or as fulfilling special ecological functions due to the strategies they have generated to adapt to these hostile environments [149,150,151,152].

The various genera identified in this study demonstrate the microbial complexity that develops in extreme environments, such as mine tailings, even with low nutrient availability and high contents of metals, such as Cu and Fe. The bacterial genera described in this study improve our understanding of the functioning of these environments, which can help develop strategies for future bioremediation of these sites (Table S1).

### 4.5. Conservation, Biodiversity, and Remediation

Cu mine tailings are a natural source of heavy-metal-tolerant bacteria with potential bioremediation capabilities [2]. These areas provide protected refuge for wildlife and reservoirs of species inhabiting extreme environments, allowing for the recovery of contaminated areas [27]. Tailing dumps contain unique microorganisms that have not yet been identified [33]. In this study, we observed that although bacterial diversity adapted to the specific conditions of each of the sampling points, all points shared a high content of various metals and exhibited similar bacterial diversity. Genetic engineering of extremophilic bacteria in stressed environments can help bioremediate contaminated sites [33].

The mechanisms by which bacteria release metal ions are varied and depend on each bacterium and environmental conditions [153]. Among these mechanisms are bioleaching (oxidation of organic compounds or sulfides), where, for example, the bacteria *Acidithiobacillus ferroxidans* oxidizes iron sulfide (pyrite) into iron sulfate and sulfuric acid, releasing metal ions in the process [154], and bacteria that can oxidize sulfides (such as chalcopyrite) release Cu^2+^ ions or other metals [155].

There is also the possibility of reducing metals. The best-known bacteria that use this mechanism are sulfate-reducing bacteria, which can reduce sulfates to sulfides, thus precipitating metals as metallic sulfides [156]. Certain heterotrophic bacteria have the ability to produce organic acids during the decomposition of organic matter, which can chelate metals, thus increasing their solubility and availability [157]. *Bacillus* has stood out as a phosphate-solubilizer that in turn could be used as a potential biofertilizer in areas with nutrient deficiencies [158]. Through the adsorption or bioaccumulation of metals, bacteria have been able to mobilize metal ions or accumulate them within themselves, which could be useful for the remediation and recovery of valuable metals [94]. In this study, it was possible to identify bacteria that have previously been reported as remediators of mine tailings (*Lysinibacillus*) [149] or that have been reported in these environments and could have a potential use in the remediation of these sites due to the strategies they have used to adapt to these conditions (*Pirellula*) [151].

Another mechanism that bacteria have to chelate Fe or other metals is the generation of siderophores, which are organic compounds produced by bacteria that have the ability to chelate metals, such as iron, thus increasing their solubility and availability for other microorganisms [159]. The genera *Bacillus* and *Staphylococcus* generate siderophores, which allows them to chelate the metals that are around them [160,161]. Both genera were identified in our study area, which, given their previously reported characteristics, would allow us to understand the possible function and role that these bacteria play in these environments.

A previous study [92] reported that the bacterium *P. denitrificans* generates a compound that can acquire Cu ions in soil with a low content of this metal. This dependent relationship between *Paracoccus* and Cu content was observed through a correlation analysis. For total Fe, total Cu, total Mo, and available Cr, a positive correlation was found, whereas for Cd both total and available concentrations showed a slightly negative correlation. Identifying how these bacteria develop mechanisms to survive in extreme conditions is useful when proposing potential remedial strategies.

Owing to their unique characteristics, these organisms and their enzymes are expected to bridge the gap between biological and chemical industrial processes [22]. However, the structural and biochemical properties of extremophilic bacteria, along with the possible long-term effects of their application, require further investigation [25].

## 5. Conclusions

Copper-mining tailings are characterized by high concentrations of heavy metals and a neutral to slightly acidic pH, depending on the process used to extract Cu, creating conditions that necessitate extreme adaptation for any organism. In this study, samples from three different sectors of the Ovejería Tailings Dam belonging to the copper mining company CODELCO were evaluated to investigate the extreme environment under which extremophile bacteria survive. Sequencing of the V3–V4 hypervariable regions of the 16S rRNA gene led to the identification of 12 phyla, 18 classes, 43 orders, 82 families, and 154 genera of bacteria at the three mining-tailing sampling sites. The phylum Proteobacteria was the most dominant, followed by Chlamydiota, Bacteroidetes, Actinobacteria, and Firmicutes. Genera, such as *Bradyrhizobium*, *Aquabacterium*, *Paracoccus*, *Caulobacter*, *Azospira*, and *Neochlamydia*, showed relatively high abundances. A chemical analysis of the tailing samples revealed high concentrations of metals, such as Fe (>30,000 mg kg^−1^) and Cu (>2000 mg kg^−1^), in addition to the presence of Cr, Cd, Mn, Mo, and Pb. *Chlamydiales* were the only intracellular bacteria exclusive to eukaryotic cells. The discovery of the genus *Neochlamydia*, which has high tolerance to toxic heavy metals, suggests an environmental reservoir of unknown ecological importance. Identifying bacteria of the *Paracoccus* genus also provided insight into the types of bacteria that can be found in such hostile environments, where their known associations with sulfates, nitrates, and high Cu contents offer insight into the complex organisms that develop in this environment.

This study represents the first study to identify the bacteria present in the Ovejería Tailings Dam, where, in turn, a point on the tailings beach located within the wall of the tailings dam was chemically characterized for the first time. These tailings belong to an active tailings dam, which is scheduled to close in 2060, which represents an opportunity to monitor the microbial communities that develop in this environment and how they can vary over time and as a result of the chemical processes that occur there. Identifying the bacteria that can inhabit environments like mine tailings is a crucial step towards better understanding these environments and developing potential bioremediation strategies.

Future research should focus on long-term monitoring of microbial communities and their adaptive mechanisms in response to changing environmental conditions, as well as exploring the potential for biotechnological applications of these extremophiles in bioremediation and other industrial processes. This study contributes significantly to the field by providing a comprehensive baseline of microbial diversity in copper-mining tailings, highlighting the resilience and adaptability of extremophiles in harsh environments. It also opens new avenues for the investigation of bioremediation strategies and the ecological roles of these microorganisms, thus enriching the scientific literature with valuable insights into microbial ecology and environmental biotechnology.

## Figures and Tables

**Table 1:** Chemical characterization of mine tailings of the Ovejería Dam (values are average ± standard deviation, *n* = 3).

**Total Metals (mg kg^−1^)**	**P1**	**P2**	**P3**
Cd	0.66 ± 0.04 a	0.63 ± 0.03 a	0.65 ± 0.09 a
Cr	13.2 ± 1.62 a	7.18 ± 0.53 b	8.09 ± 1.17 b
Cu	2488 ± 258 a	2986 ± 102 a	2911 ± 806 a
Fe	35,960 ± 3680 a	26,583 ± 2508 b	34,604 ± 2156 a
Mn	185.8 ± 20.3 a	236.2 ± 15.7 b	268.6 ± 18.1 b
Mo	145.6 ± 8.90 a	268.8 ± 84.1 a	169.03 ± 21.8 a
Pb	9.53 ± 0.73 a	8.63 ± 1.33 a	11.62 ± 3.56 a
**Available Metals (DTPA) (mg kg^−1^)**	**P1**	**P2**	**P3**
Cd	0.032 ± 0.002 a	0.029 ± 0.003 a	0.033 ± 0.001 a
Cr	0.193 ± 0.014 a	0.211 ± 0.012 a	0.206 ± 0.011 a
Cu	67.1 ± 2.9 a	69.8 ± 4.1 a	80.4 ± 4.3 b
Fe	7.34 ± 0.32 a	6.3 ± 0.82 a	7.13 ± 0.8 a
Mn	6.69 ± 0.2 a	1.25 ± 0.08 b	10.5 ± 1.14 c
Mo	nd	0.55 ± 0.07	nd
Pb	0.16 ± 0.02 a	0.25 ± 0.02 ab	0.40 ± 0.1 b
**Other Variables**	**P1**	**P2**	**P3**
pH	6.70	6.85	6.20
Electrical conductivity (dS m^−1^)	4.08	2.22	2.11
SO_4_^−^(mmol/L)	16.75	2.11	3.05

Abbreviations: Cd, cadmium; Cr, chromium; Cu, copper; Fe, iron; Mn, manganese; Mo, molybdenum; Pb, lead; DTPA, diethylenetriaminepentaacetic acid; nd, not detected. Equal letters next to the standard deviation mean there are no significant differences; different letters indicate significant differences.

## Data Availability

All raw sequences were deposited in the National Center for Biotechnology Information (NCBI; Bethesda, MD, USA) database (BioProject accession number: PRJNA1143042).

## Supplementary Materials

The following supporting information can be downloaded at https://www.mdpi.com/article/10.3390/microorganisms12091820/s1, Data S1: Relative abundances of the 10 most abundant genera (highest mean relative abundance) of each sample site in the copper mine Ovejería Tailings Dam. Table S1: Relative abundance (%) of the genera identified at the different sampled points.
