# Supplementary material for: Profile of Bacterial Communities in Copper Mine Tailings Revealed through High-Throughput Sequencing
Source: Microorganisms. 2024 Sep 3;12(9):1820. doi: 10.3390/microorganisms12091820 (PMC11433839; doi:10.3390/microorganisms12091820)
Supplement: Supplementary file 1 [file microorganisms-12-01820-s001.zip › microorganisms-3166628-supplementary.pdf]

Supplemental Data

Data S1: Relative abundances of the 10 most abundant genera (highest mean relative abundance) of each sample site in copper mine Ovejería tailings dam

Phylum: Chlamydiota- Class: Chlamydiia

- Order: *Chlamydiales* - Family: *Parachlamydiaceae*

Neochlamydia This genus has only its type strain (*Neochlamydia hartmannellae*) taxonomically described, twelve unclassified isolates and a few non-cultured environmental strains. They are obligate intracellular bacteria of free-living amoebae. They depend on a eukaryotic cell for their replication. They are found in aquatic environments and soils and may be emerging pathogens. They could influence the biotransformation of organic and inorganic compounds in mine tailings, as well as being able to tolerate high concentrations of metals [1,2].

Phylum: Pseudomonadota. Class: Alphaproteobacteria

- Order: *Hyphomicrobiales* - Family: *Nitrobacteraceae*

Bradyrhizobium. Approximately 92 species have been described. Slow-growing bacteria form symbiotic relationships with leguminous plants, fixing atmospheric nitrogen into forms usable by plants. They are producers of indoleacetic acid (IAA), which is a plant growth hormone and, in addition to their ability to tolerate metals and certain concentrations of NaCl, could help in phytoremediation by improving soil fertility in mine tailings [2-11].

- Order: *Caulobacterales* - Family: *Caulobacteraceae*

Caulobacter Are commonly found in aquatic and soil ecosystems, are oligotrophic, aerobic and Gram-negative bacteria, of which 27 species have been described taxonomically. They are known for their asymmetric cell cycle and their role in cell cycle regulation. They are known to solubilize phosphate and produce IAA. They could be involved in the bioprecipitation of heavy metals in tailings [12-17].

- Order: *Rhodobacterales* - Family: *Paracoccaceae*

Paracoccus Has approximately 108 described species. Ubiquitous bacteria in terrestrial and aquatic environments. They participate in the nitrogen cycle, being able to perform aerobic denitrification and reduce Cu concentrations. In mine tailings, they could contribute to the nitrogen cycle and help in the bioremediation of metals [1,16-19].

Phylum: Pseudomonadota. Class: Betaproteobacteria

- Order: *Burkholderiales* - Family: *Comamonadaceae*

Aquabacterium Aerobic Gram-negative bacteria isolated from biofilms in drinking water systems, of which 12 species have been taxonomically described. They participate in the degradation of organic compounds in aquatic environments, being able to oxidize and denitrify Fe. There is little information on their relationship with metals, but their ability to withstand concentrations of Cd, Cu, Fe and Pb has been observed [11,19-21].

- Order: *Rhodocyclales* - Family: *Rhodocyclaceae*

Azospira Only four species of this genus have been taxonomically described. This genus is a nitrogen-fixing bacteria that can grow anaerobically by reducing perchlorate. The effect of copper on its functioning as aniline degraders and on its interactions with nitrogen has been studied, where a decrease in its efficiency was observed. They promote plant growth and resistance to diseases [22,23].

Phylum: Bacteroidota. Class: Bacteroidia

- Order: *Bacteroidales* - Family: *Prevotellaceae*

Prevotella Aproximatelly 78 species of Prevotella have been described. They have been found to be anaerobic bacteria. carbohydrates, producing succinate and acetate. They are found in the oral cavity and the intestinal tract. Species with the ability to tolerate metals such as Cd, Cr, Cu, Fe and Zn have been described. They can participate in the degradation of organic matter or biodiesel [24].

Phylum: Bacteroidota. Class: Flavobacteriia

- Order: *Flavobacteriales* - Family: *Weeksellaceae*

Chryseobacterium Have more than 170 described species. Gram-negative bacteria found in soil, freshwater and marine environments. Some species can inhibit phytopathogenic fungi. It is capable of producing IAA and tolerating high concentrations of Cd and Cu. In addition, its resistance to antibiotics has been studied since certain species can cause human infections [6,8-9,25-28].

Phylum: Firmicutes. Class: Bacilli

- Order: *Caryophanales* - Family: *Staphylococcaceae*

Staphylococcus At least 89 species have been described. Gram-positive bacteria that inhabit the skin and mucous membranes of humans and animals. Some species are pathogenic and cause nosocomial infections. The species *S. lentus* is capable of generating biofilms and has the ability to tolerate high concentrations of Cu2+ ions. Certain species can be pathogenic in the human urinary tract [29-31].

Phylum: Actinomycetota. Class: Actinomycetia

- Order: *Propionibacteriales* - Family: *Propionibacteriaceae*

Cutibacterium Only seven species has been described of this genus. Gram-positive bacteria that inhabit human skin, especially in areas rich in sebum. They contribute to skin homeostasis and may be involved in acne. The species *C. acnes* has been described together with *Staphylococcus epidermis*, which are capable of generating biofilms and tolerating high concentrations of Cu, Pb, NaCl (50 g) and an alkaline pH. In turn, their response to the degradation of hydrocarbons and metals has been studied [32-35].

References

[1] Horn, M.; Wagner, M. Evidence for additional genus-level diversity of Chlamydiales in the environment. *FEMS Microbiol Lett* **2001**, *204*(1), 71–74, doi:10.1111/j.1574-6968.2001.tb10865.x.

[2] Gerrity, D.; Arnold, M.; Dickenson, E.; Moser, D.; Sackett, J.D.; Wert, E.C. Microbial community characterization of ozone biofiltration systems in drinking water and potable reuse applications. *Water Res* **2018**, *135*, 207–219. doi:10.1016/j.watres.2018.02.023.

[3] Dary, M.; Chamber-Pérez, M.A.; Palomares, A.J.; Pajuelo, E. "In situ" phytostabilisation of heavy metal polluted soils using Lupinus luteus inoculated with metal resistant plant-growth promoting rhizobacteria. *J Hazard Mater* **2010**, *177*(1-3), 323–330. doi:10.1016/j.jhazmat.2009.12.035

[4] Salmi, A.; Boulila, F. Heavy metals multi-tolerant Bradyrhizobium isolated from mercury mining region in Algeria. *J Environ Manage* **2021**, *289*, 112547. doi:10.1016/j.jenvman.2021.112547

[5] VanInsberghe, D.; Maas, K. R.; Cardenas, E.; Strachan, C. R.; Hallam, S. J.; Mohn, W. W. Non-symbiotic Bradyrhizobium ecotypes dominate North American forest soils. *ISME J* **2015**, *9*(11), 2435–2441. doi:10.1038/ismej.2015.54

[6] Zhong, C.; Hu, G.; Hu, C.; Xu, C.; Zhang, Z.; Ning, K. Comparative genomics analysis reveals genetic characteristics and nitrogen fixation profile of *Bradyrhizobium*. *iScience* **2024**, *27*(2), 108948. doi:10.1016/j.isci.2024.108948

[7] Seneviratne, M.; Gunaratne, S.; Bandara, T.; Weerasundara, L.; Rajakaruna, N.; Seneviratne, G.; Vithanage, M. Plant growth promotion by Bradyrhizobium japonicum under heavy metal stress. *S Afr J Bot* **2016**, *105*, 19–24. doi:10.1016/j.sajb.2016.02.206

[8] Mano, Y.; Nemoto, K. The pathway of auxin biosynthesis in plants. *J Exp Bot* **2012**, *63*, 2853–2872. doi:10.1093/jxb/ers091

[9] Glick, B.R.; Karaturovíc, D.M.; Newell, P.C. A novel procedure for rapid isolation of plant growth promoting pseudomonads. *Can J Microbiol* **1995**, *41*, 533–536. doi:10.1139/m95-070

[10] Glick, B.R.; Todorovic, B.; Czarny, J.; Cheng, Z.; Duan, J.; McConkey, B. Promotion of plant growth by bacterial ACC deaminase. *Crit Rev Plant Sci* **2007**, *26*, 227–242. doi:10.1080/07352680701572966

[11] Xu, L.; Su, J.; Li, K.; Hu, R.; Yan, H.; Liang, E.; Zhou, Z.; Shi, J. Performance of hydrogel-immobilized bioreactors combined with different iron ore wastes for denitrification and removal of copper and lead: optimization and possible mechanism. *Water Res* **2022**, *225*, 119196. doi:10.1016/j.watres.2022.119196.

[12] Benyehuda, G.; Coombs, J.; Ward, P.L.; Balkwill, D.; Barkay, T. Metal resistance among aerobic chemoheterotrophic bacteria from the deep terrestrial subsurface. Can J Microbiol **2003**, 49, 151–156. doi:10.1139/w03-012

[13] Inagaki, F.; Takai, K.; Hirayama, H.; Yamato, Y.; Nealson, K.H.; Horikoshi, K. Distribution and phylogenetic diversity of the subsurface microbial community in a Japanese epithermal gold mine. Extremophiles **2003**, 7, 307–317. doi:10.1007/s00792-003-0324-9

[14] North, N.N.; Dollhopf, S.L.; Petrie, L.; Istok, J.D.; Balkwill, D.L.; Kostka, J.E. Changes in bacterial community structure during in situ biostimulation of subsurface sediment contaminated with uranium and nitrate. *Appl Environ Microbiol* **2004**, *70*, 4911–4920. doi:10.1128/AEM.70.8.4911-4920.2004

[15] Maertens, L.; Cherry, P.; Tilquin, F.; Van Houdt, R.; Matroule, J.-Y. Environmental conditions modulate the transcriptomic responses of both Caulobacter crescentus morphotypes to Cu stress. *Microorganisms* **2021**, *9*, 1116. doi:10.3390/microorganisms9061116.

[16] Andreazza, R.; Okeke, B.C.; Pieniz, S.; Bortolon, L., Lambais, M. R.,; Camargo, F. A. Effects of Stimulation of Copper bioleaching on microbial community in vineyard soil and Copper mining waste. *Biol Trace Elem Res* **2012**, *146*, 124–133. doi:10.1007/s12011-011-9213-8.

[17] Stouthamer, A.H. Metabolic regulation, including anaerobic metabolism in Paracoccus denitrificans. *J Bioenerg Biomembr* **1991**, *23*, 163–185. doi:10.1007/BF00762216

[18] Anttila, J.; Heinonen, P.; Nenonen, T.; Pino, A.; Iwaï, H.; Kauppi, E.; Soliymani, R.; Baumann, M.; Saksi, J.; Suni, N.; Haltia, T. Is coproporphyrin III a copper acquisition compound in Paracoccus denitrificans? *Biochimica et Biophysica Acta (BBA) – Bioenergetics* **2011**, *1807*(3), 311–318. doi:10.1016/j.bbabio.2010.12.014

[19] Lu, Z.; Gan, L.; Lin, J.; Chen, Z. Aerobic denitrification by Paracoccus sp. YF1 in the presence of Cu(II). Sci Total Environ 2018, 658, 80-86. doi:10.1016/j.scitotenv.2018.12.225

[20] Zhao, Y.; Gao, J.; Wang, Z.; Cui, Y.; Zhang, Y.; Dai, H.; Li, D. Distinct bacterial communities and resistance genes enriched by triclocarban-contaminated polyethylene microplastics in antibiotics and heavy metals polluted sewage environment. *Sci Total Environ* **2022**, *839*, 156330. doi:10.1016/j.scitotenv.2022.156330

[21] Kroeksakul, P.; Ngamniyom, A.; Silprasit, K.; Sutthisaksopon, P.; Sriyapai, T.; Phowan, N.; Singhaboot, P. Evaluation of Pesticide and Heavy Metal Contamination on Soil Properties and Microbiota in Thailand's mountainous region. *J Ecol Eng* **2023**, *24*(7), 331-344. doi:10.12911/22998993/165957.

[22] Su, J.; Zhang, Q.; Peng, H.; Feng, J.; He, J.; Zhang, Y.; Lin, B.; Wu, N.; Xiang, Y. Exploring the impact of intensity and duration of Cu (II) depression on aniline-degrading biosystem: Performance, sludge activity, and microbial diversity. *Bioresour Technol* **2022**, *360*, 127548. doi:10.1016/j.biortech.2022.127548.

[23] Shi, X.; Duan, Z.; Wang, J.; Zhou, W.; Jiang, M.; Li, T.; Ma, H.; Zhu, X. Simultaneous removal of multiple heavy metals using single-chamber microbial electrolysis cells with biocathode in the microaerobic environment. *Chemosphere* **2023**, *318*, 137982. doi:10.1016/j.chemosphere.2023.137982.

[24] Salam, N., Jiao, J. Y., Zhang, X. T., & Li, W. J. Update on the classification of higher ranks in the phylum Actinobacteria. *International Journal Of Systematic And Evolutionary Microbiology.* **2020**, 70(2), 1331–1355. doi:10.1099/ijsem.0.003920

[25] Bernardet, J.F.; Hugo, C.; Bruun, B. (2006). The Genera: Chryseobacterium and Elizabethkingia. In: Dworkin, M., Falkow, S., Rosenberg, E., Schleifer, KH., Stackebrandt, E. (eds) The Prokaryotes. Springer, New York, NY, USA. doi:10.1007/0-387-30747-8_25.

[26] Majewska, M.; Wdowiak-Wróbel, S.; Marek-Kozaczuk, M.; *et al*. Cadmium-resistant Chryseobacterium sp. DEMBc1 strain: characteristics and potential to assist phytoremediation and promote plant growth. Environ Sci Pollut Res **2022**, 29, 83567–83579. doi:10.1007/s11356-022-21574-3.

[27] Glibota, N., Grande Burgos, M.J.; Gálvez, A.; Ortega, E. Copper tolerance and antibiotic resistance in soil bacteria from olive tree agricultural fields routinely treated with copper compounds. *J Sci Food Agric* **2019**, *99*(10), 4677–4685. doi:10.1002/jsfa.9708

[28] Yang, E.; Sun, L.; Ding, X.; Sun, D.; Liu, J.; Wang, W. Complete genome sequence of Caulobacter flavus RHGG3T, a type species of the genus Caulobacter with plant growth-promoting traits and heavy metal resistance. *3 Biotech* **2019**, *9*, 42. doi:10.1007/s13205-019-1569-z.

[29] Lawal, O.U.; Fraqueza, M.J.; Worning, P.; Bouchami, O.; Bartels, M.D.; Goncalves, L.; Paixão, P.; Goncalves, E.; Toscano, C.; Empel, J.; Urbaś, M.; Domínguez, M.A.; Westh, H.; de Lencastre, H.; Miragaia, M. Staphylococcus saprophyticus Causing Infections in Humans Is Associated with High Resistance to Heavy Metals. *Antimicrob Agents Chemother* **2021**, *65*(7), 10-1128. doi:10.1128/aac.02685-20.

[30] Padmavathi, A.R.; Sriyutha Murthy, P.; Das, A.; Nishad, P.A.; Pandian, R.; Rao, T.S. Copper oxide nanoparticles as an effective anti-biofilm agent against a copper tolerant marine bacterium, Staphylococcus lentus. *Biofouling* **2019**, *35*(9), 1007–1025. doi:10.1080/08927014.2019.1687689.

[31] Zapotoczna, M.; Riboldi, G.P.; Moustafa, A.M.; Dickson, E.; Narechania, A.; Morrissey, J.A.; Planet, P.J.; Holden, M.T.G.; Waldron, K.J.; Geoghegan, J.A. Mobile-Genetic-Element-Encoded Hypertolerance to Copper Protects Staphylococcus aureus from Killing by Host Phagocytes. *MBio* **2018**, *9*(5), 10-1128. doi:10.1128/mbio.00550-18.

[32] Huang, S.; Chen, C.; Jaffé, P.R. Seasonal distribution of nitrifiers and denitrifiers in urban river sediments affected by agricultural activities. *Sci Total Environ* **2018**, *642*, 1282–1291. doi:10.1016/j.scitotenv.2018.06.116.

[33] Zhang, S.-Y.; Tsementzi, D.; Hatt, J.K.; Bivins, A.; Khelurkar, N.; Brown, J.; Tripathi, S. N.; Konstantinidis, K. T. Intensive allochthonous inputs along the Ganges River and their effect on microbial community composition and dynamics. *Environ Microbiol* **2019**, *21*, 182–196. doi:10.1111/1462-2920.14439.

[34] Godoy, R.G.; Marcondes, M.A.; Pessôa, R.; Nascimento, A.; Victor, J. R.; Duarte, A. J. D. S.; Clissa, P. B.; Sanabani, S. S. Bacterial community composition and potential pathogens along the Pinheiros River in the southeast of Brazil. *Sci Rep* **2020**, *10*, 1–9. doi:10.1038/s41598-020-66386-y.

[35] Lenchi, N.; Ahmedi, W.N.E.H.; Llirós, M. Simultaneous removal of crude oil and heavy metals by highly adapted bacterial strain Cutibacterium sp. NL2 isolated from Algerian oilfield. *Int Microbiol* **2024**, *27*, 615–630. doi:10.1007/s10123-023-00419-0.

Table S1. Relative abundance (%) of the genera identified at the different sampled points.

N	Genus	Mean Relative Abundance (%)		
		P1	P2	P3
1	*Neochlamydia*	43.838	27.900	36.689
2	*Bradyrhizobium*	23.537	34.437	24.692
3	*Aquabacterium*	6.557	3.985	8.820
4	*Chryseobacterium*	7.300	5.178	6.311
5	*Caulobacter*	5.900	3.780	7.595
6	*Staphylococcus*	2.675	1.632	0.776
7	*Paracoccus*	2.612	0.908	0.209
8	*Cutibacterium*	1.386	0.995	0.341
9	*Prevotella 9*	0.000	0.180	2.303
10	*Azospira*	0.618	0.767	0.935
11	*Escherichia–Shigella*	0.386	1.067	0.728
12	*Bacteroides*	0.000	1.196	0.631
13	*Curvibacter*	0.568	0.076	0.965
14	*Corynebacterium 1*	0.344	0.211	1.008
15	*Flavobacterium*	0.190	1.268	0.057
16	*Bosea*	0.211	0.678	0.215
17	*Lactobacillus*	0.000	0.602	0.433
18	*Kocuria*	0.158	0.584	0.282
19	*Empedobacter*	0.028	0.872	0.000
20	*Cupriavidus*	0.312	0.270	0.221
21	*Lawsonella*	0.284	0.315	0.149
22	*Lachnospiraceae UCG-004*	0.000	0.629	0.000
23	*Streptococcus*	0.211	0.315	0.097
24	*Mesorhizobium*	0.257	0.000	0.365
25	*Hyphomicrobium*	0.339	0.057	0.163
26	*Alistipes*	0.000	0.539	0.000
27	*Reyranella*	0.053	0.216	0.256
28	*Tetrasphaera*	0.000	0.521	0.000
29	*Methylobacterium*	0.290	0.023	0.179
30	*Lysinibacillus*	0.000	0.477	0.000
31	*Cylindrospermopsis CRJ1*	0.000	0.459	0.000
32	*Ferruginibacter*	0.000	0.000	0.448
33	*Asticcacaulis*	0.198	0.113	0.127
34	*Prosthecobacter*	0.000	0.405	0.000
35	*Devosia*	0.195	0.117	0.090
36	*Pirellula*	0.000	0.396	0.000
37	*Planomicrobium*	0.000	0.387	0.000
38	*Pullulanibacillus*	0.000	0.387	0.000
39	*Prevotella 7*	0.000	0.378	0.000
40	*Methylovirgula*	0.000	0.369	0.000
41	*Aphanizomenon MDT14a*	0.000	0.360	0.000
42	*Synechococcus CC9902*	0.000	0.360	0.000
43	*Thiobacillus*	0.142	0.000	0.208
44	*Fimbriiglobus*	0.179	0.038	0.117
45	*Holdemanella*	0.000	0.000	0.328
46	*Roseburia*	0.000	0.306	0.018
47	*Woeseia*	0.000	0.324	0.000
48	*Butyricicoccus*	0.000	0.234	0.084
49	*Ruminococcus 2*	0.000	0.306	0.012
50	*Blautia*	0.000	0.000	0.307
51	*Blastococcus*	0.000	0.288	0.000
52	*Pir2 lineage*	0.000	0.288	0.000
53	*Micrococcus*	0.000	0.227	0.057
54	*Lachnospiraceae NK4A136* group	0.000	0.279	0.000
55	*Ruminococcaceae UCG-014*	0.000	0.000	0.277
56	*Arcanobacterium*	0.000	0.270	0.000
57	*Chthoniobacter*	0.000	0.270	0.000

N	Genus	Mean Relative Abundance (%)		
		P1	P2	P3
58	*Defluviicoccus*	0.000	0.270	0.000
59	*Rhodocytophaga*	0.000	0.270	0.000
60	*Aphanizomenon NIES81*	0.000	0.261	0.000
61	*Subdoligranulum*	0.000	0.000	0.240
62	*Sporichthya*	0.000	0.225	0.000
63	*Methyloversatilis*	0.000	0.189	0.024
64	*Bacillus*	0.140	0.072	0.000
65	*Marmoricola*	0.000	0.090	0.119
66	*Brachybacterium*	0.000	0.207	0.000
67	*Microbacterium*	0.038	0.064	0.103
68	*Nodosilinea PCC-7104*	0.000	0.198	0.000
69	*Limnobacter*	0.000	0.189	0.000
70	*Enterobacter*	0.137	0.034	0.014
71	*Fibrobacter*	0.000	0.171	0.000
72	*Legionella*	0.000	0.000	0.168
73	*Edaphobacter*	0.038	0.076	0.053
74	*Antricoccus*	0.000	0.162	0.000
75	*Allorhizobium–Neorhizobium–Pararhizobium–Rhizobium*	0.081	0.000	0.073
76	*Cyanobium PCC-6307*	0.000	0.153	0.000
77	*Peptoniphilus*	0.116	0.000	0.030
78	*Fusicatenibacter*	0.000	0.000	0.144
79	*Mycobacterium*	0.000	0.144	0.000
80	*Prevotella*	0.000	0.144	0.000
81	*Sulfurifustis*	0.000	0.000	0.132
82	*Alloprevotella*	0.000	0.000	0.129
83	*Clostridium sensu stricto 1*	0.000	0.000	0.126
84	*Faecalibacterium*	0.000	0.000	0.126
85	*Coprococcus 3*	0.000	0.000	0.123
86	*Nubsella*	0.042	0.000	0.079
87	*Jeotgalibacillus*	0.000	0.117	0.000
88	*Gardnerella*	0.000	0.000	0.117
89	*Peptoclostridium*	0.000	0.000	0.114
90	*Massilia*	0.042	0.000	0.068
91	*Abiotrophia*	0.105	0.000	0.000
92	*Gemella*	0.105	0.000	0.000
93	*Eremococcus*	0.000	0.099	0.000
94	*Filifactor*	0.000	0.099	0.000
95	*Rothia*	0.000	0.099	0.000
96	*Sutterella*	0.000	0.000	0.093
97	*Clostridium sensu stricto 8*	0.000	0.000	0.084
98	*Pantoea*	0.000	0.054	0.028
99	*Ramlibacter*	0.000	0.000	0.079
100	*Deinococcus*	0.042	0.034	0.000
101	*Pseudohongiella*	0.000	0.000	0.072
102	*Alicyclobacillus*	0.000	0.072	0.000
103	*Ruminococcaceae UCG-010*	0.000	0.000	0.066
104	*Finegoldia*	0.053	0.000	0.011
105	*Pediococcus*	0.063	0.000	0.000
106	*Pontibacter*	0.063	0.000	0.000
107	*Segetibacter*	0.000	0.000	0.057
108	*Neisseria*	0.000	0.000	0.056
109	*Chryseomicrobium*	0.000	0.000	0.055
110	*Acidipila*	0.000	0.054	0.000
111	*Fusobacterium*	0.000	0.054	0.000
112	*Luteolibacter*	0.000	0.054	0.000
113	*Carnobacterium*	0.053	0.000	0.000
114	*Geomicrobium*	0.000	0.000	0.049
115	*Terrimicrobium*	0.031	0.000	0.018
116	*Salinimicrobium*	0.000	0.000	0.048

N	Genus	Mean Relative Abundance (%)		
		P1	P2	P3
117	*Paenibacillus*	0.000	0.000	0.045
118	*Lautropia*	0.000	0.000	0.045
119	*Gracilibacter*	0.000	0.045	0.000
120	*Haemophilus*	0.000	0.045	0.000
121	*Rubrobacter*	0.042	0.000	0.000
122	*Adhaeribacter*	0.000	0.000	0.042
123	*Jeotgalicoccus*	0.000	0.000	0.039
124	*Leptotrichia*	0.000	0.000	0.039
125	*Gemmata*	0.000	0.000	0.035
126	*Ornithinimicrobium*	0.000	0.000	0.035
127	*Bifidobacterium*	0.000	0.000	0.033
128	*Modestobacter*	0.000	0.000	0.033
129	*[Eubacterium] hallii* group	0.000	0.000	0.030
130	*Romboutsia*	0.000	0.000	0.030
131	*Tyzzerella*	0.000	0.000	0.030
132	*Virgibacillus*	0.000	0.000	0.030
133	*Aggregatibacter*	0.000	0.000	0.027
134	*Leifsonia*	0.000	0.000	0.027
135	*Facklamia*	0.000	0.026	0.000
136	*Odoribacter*	0.000	0.000	0.024
137	*Olsenella*	0.000	0.000	0.024
138	*Hymenobacter*	0.021	0.000	0.000
139	*Hydrogenispora*	0.000	0.000	0.021
140	*Actinomyces*	0.000	0.000	0.021
141	*Tumebacillus*	0.000	0.000	0.021
142	*Nocardioides*	0.019	0.000	0.000
143	*[Ruminococcus] torques* group	0.000	0.000	0.018
144	*Ochrobactrum*	0.000	0.000	0.018
145	*Brevibacterium*	0.000	0.000	0.018
146	*Pajaroellobacter*	0.000	0.000	0.016
147	*Actinobacillus*	0.000	0.000	0.015
148	*Haliangium*	0.000	0.000	0.015
149	*Meiothermus*	0.000	0.000	0.015
150	*Noviherbaspirillum*	0.000	0.000	0.015
151	*Pedomicrobium*	0.000	0.000	0.014
152	*Snowella 0TU37S04*	0.000	0.000	0.014
153	*Enterococcus*	0.000	0.000	0.012
154	*Negativibacillus*	0.000	0.000	0.006
